# Augmented Reality (AR) in Surgery in Low- and Middle-Income Countries (LMICs): A Scoping Review

**DOI:** 10.7759/cureus.64278

**Published:** 2024-07-10

**Authors:** Vania Arboleda, Aryan Lajevardi, Pierina Barletti, Mariapia Medina, Apurva Ramanujam, Kawther N Elsouri, Michelle Demory

**Affiliations:** 1 Osteopathic Medicine, Nova Southeastern University Dr. Kiran C. Patel College of Osteopathic Medicine, Fort Lauderdale, USA; 2 Biomedical Sciences, Nova Southeastern University, Orlando, USA; 3 Immunology, Nova Southeastern University Dr. Kiran C. Patel College of Allopathic Medicine, Fort Lauderdale, USA

**Keywords:** low- and middle-income countries, global surgery partnerships, surgical navigation systems, augmented reality (ar), global surgical access

## Abstract

Surgical disparities persist in low- and middle-income countries (LMICs). Insufficient access to surgical care places a large burden on these regions, with high mortality rates for otherwise standard procedures performed in high-income countries (HICs). Augmented Reality (AR) and Virtual Reality (VR) now provide us with a platform to improve the delivery of surgical access and training to LMICs. The use of AR technologies to provide additional training to surgeons and residents globally can help bridge the gap and reduce health disparities in LMICs. The goal of this scoping review is to evaluate whether surgical trainees and surgeons from LMICs have access to or use AR software in their training or practice. A systematic search was conducted on seven databases. Inclusion criteria included populations in LMICs with access to AR-based training. Articles using VR software, or those conducted in HICs were excluded from the review. From the 428 records screened, 58 reports were assessed for eligibility, and of these, a total of six studies were included in the review. Five of the six studies used mentors from an HIC, including the United States (US) and the United Kingdom (UK), whereas one study had mentorship from another LMIC. Three surgical specialties were explored: neurosurgery, plastic surgery, and urology. Although the integration of AR in surgical training is promising, the six studies evaluated in this review emphasize that costs and connection issues are major challenges that can set back these technologies in the operating room. Despite these revelations, with certain improvements, AR training programs are promising as they can help to reduce the global disparity in surgical proficiency.

## Introduction and background

Substantial inequalities in access to quality health services and health outcomes persist in low- and middle-income countries (LMICs) [1]. Among the most significant health disparities is the limited access to quality surgical resources in LMICs [2]. Annually, surgical disease is associated with approximately seven million deaths (10.4%), accounting for 14.2% of all disability-adjusted life years in LMICs [3]. The scarcity of surgical access in LMICs suggests a significant worldwide disease burden. Out of the necessary surgical operations required around the world, only 3.5% are in LMICs, leading to mortality, disability, and impairment within the population of these countries [4]. Additionally, surgeries performed in high-income countries (HICs) with minimal morbidity and mortality, such as hernia repair and appendectomy, are the center of fatalities in LMICs due to inadequate surgical management [5]. Overall, the trend in the mortality rate, including postoperative death, continues to grow in LMICs due to insufficient surgical development [6,7].

Effective surgical care requires a significant investment in training and infrastructure [8]. Surgical management is a complex and resource-intensive environment, requiring a surgeon, anesthesiologist, and highly educated operating room team. Plus, there is a constant on-demand supply of pharmacological and surgical instruments [9]. Further, it is necessary to have a continuous supply of electricity, which is not guaranteed in some resource-limited rural settings [10]. Although accessibility, availability, and affordability of surgical care hinder improvements in LMICs, some believe technological interventions in these settings can be cost-effective and improve surgical care in the long run [8]. Surgical practice and training that includes Augmented Reality (AR) may present an intervention to reduce health disparities in LMICs [11].

The terms AR and Virtual reality (VR) are often used in conjunction when speaking about modern technological advances and their applications. Yet, it is vital to know the differences between both. AR is the technology that allows a viewer's accurate world perception to be overlaid with digital content simultaneously [12]. By contrast, VR is a fully digital environment that is computer-generated with no transparency to the real-world environment around the user [13]. Advancements in software algorithms in AR and VR have allowed for their use in a wide array of applications, now centered on training skilled workers [14]. Surgeons, educators, and students can access VR via dedicated headsets, ranging from entry-level devices (limited in features and requiring an outside processor such as a computer) to be as low as $50-$100 USD, and do not exceed $500 [15,16]. However, for stand-alone VR devices that do not need to rely on an external computer to function and offer more features for virtual enhancement, the price can be upwards of $1,000 USD.

In contrast, AR has more price variation when referring to a stand-alone device independent of an outside processor. AR can be as low as no additional cost if the individual already has a functioning smartphone or smart tablet that is internet accessible. Unlike VR, an AR device does not need to be specifically built or dedicated to AR for it to be able to support the technology, making it less costly for those seeking to use its applications and a more accessible resource, especially in the case of LMICs.

Despite limited resources, AR technology has enhanced surgical training and improved patient surgical management in LMICs [17]. In Gaza and Palestine, using tablets and a smartphone camera, the surgical team was able to get telesurgical instruction and live annotation from a surgeon in Beirut. The surgeon in Beirut could live-guide and annotate over the live video stream of the surgery in Gaza using AR technology. This technology allowed two-way comprehensive communication between the two surgeons and visual guidance, which positively impacted the Gaza surgical team's ability to complete this procedure [18]. AR solutions have also been proposed in the context of point-of-care ultrasound (POCUS) training and interpretation in which trainees could see, provide, and receive real-time guidance on probe placement and image interpretation, increasing the quality and availability of training in LMICs [19]. Given the ease of use and relatively low cost associated with the use of AR in healthcare, this study explores whether surgical trainees and surgeons from LMICs have access to or use AR software in their training or practice.

## Review

Methods

Study Design

**Figure 1:**
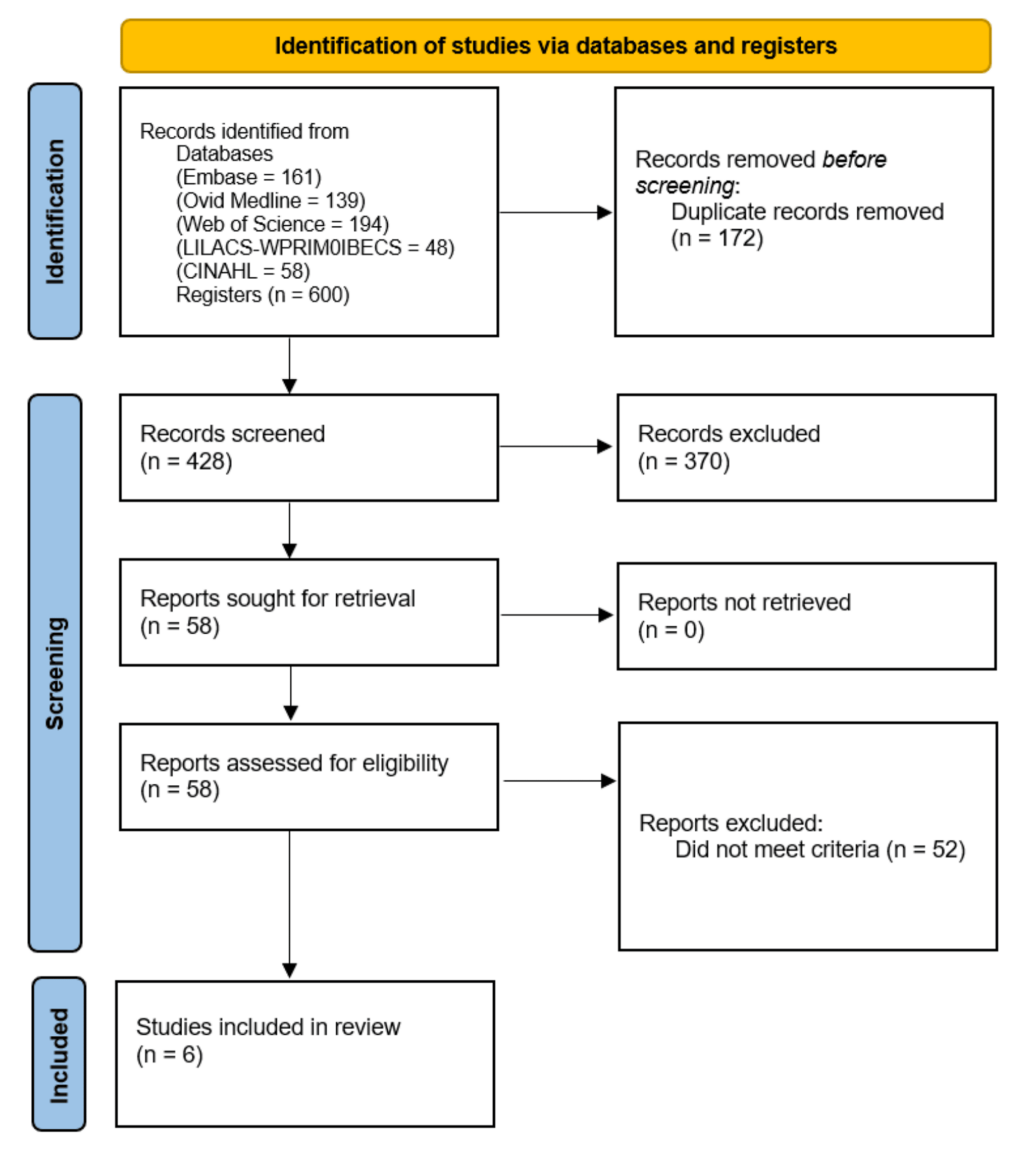
PRISMA flow chart PRISMA: Preferred Reporting Items for Systematic Reviews and Meta-Analyses

A systematic search was conducted and completed in July 2023 to evaluate whether surgical trainees and surgeons from LMICs have access to AR-based training or practice following the guidelines of the Preferred Reporting Items for Systematic Reviews and Meta-Analyses (PRISMA) (Figure 1). No funding was provided, and Institutional Review Board (IRB) and ethical approvals were not required because this review of previously published literature did not include participants' information.

Inclusion and Exclusion Criteria

The criteria to include articles followed the population, concept, context (PCC) framework. Eligible populations included surgeons and/or surgical residents/students or surgical field teams in any surgical specialty. As concept, the population subjects had to use or have access to AR-based training or practice. As context, the population subjects using the AR technology had to be from LMICs. Excluded articles were articles involving VR software, articles in HICs, articles not in Spanish or English, articles involving animal subjects, and any articles/studies not adhering to the established PCC framework.

Search Strategy and Screening

In July 2023, a search was conducted on seven databases: MEDLINE, Embase, CINAHL, LILACS, Scielo, Cochrane Library, and Web of Science. Keywords, phrases, and possible synonyms and abbreviations were combined with Boolean logic terms. Articles were filtered for matches and then exported into Rayyan, a web tool for sorting articles. Four researchers (KE, AL, PB, MM) individually sorted abstracts of articles based on the inclusion and exclusion criteria. Conflicts between each team on an abstract's inclusion and exclusion criteria were resolved by an independent researcher (VA). Next, the included abstracts were reentered as full-text articles into Rayyan and were evaluated by the five researchers based on the inclusion and exclusion criteria. Further conflicts between full-text articles were resolved by majority agreement in a 3:2 ratio in favor. All researchers conducted a final review of articles to validate their quality and adherence to eligibility criteria.

Results

The systematic search resulted in a total of 600 articles. A total of 172 duplicates were identified and removed before initiating screening. From the remaining records, 428 abstracts were screened and 370 articles did not meet the inclusion criteria or met the exclusion criteria. The resulting 58 records were sought for retrieval and assessed for eligibility. Of the 58 articles, six were included (Table 1).

**Table 1:** Summary of articles included US: United States; UK: United Kingdom

Study	Study Type	Mentor	Mentee	Specialty
Greenfield et al. 2018 [18]	Case Report	Lebanon (n =1)	Palestine (n= 1)	Plastic surgery
Davis et al. 2016 [20]	Retrospective	US	Vietnam	Neurosurgery
McCullough et al. 2018 [21]	Case Study	US (n = 1)	Mozambique (n = 1)	Plastic surgery
Vyas et al. 2020 [22]	Prospective	US	Peru (n = 2)	Plastic surgery
Sommer et al. 2022 [23]	Prospective	US	Tanzania	Neurosurgery
Dominique et al. 2023 [24]	Retrospective	US (n = 2); UK (n = 1)	Benin, Ethiopia, Nigeria, Senegal (n = 4)	Urology

Five of the six included articles [20-24] had surgeon mentors from HICs, with 5/5 surgeon mentor teams overseeing from the United States (US). Dominique et al. had a United Kingdom (UK) surgeon mentor team in addition to the American surgeon team [24] (Table 1). Greenfield et al.'s article was the only article that had an LMIC surgeon mentor from Lebanon overseeing another LMIC surgeon mentee from Palestine [18]. The mentee surgeons participating were from Vietnam, Mozambique, Palestine, Peru, Tanzania, Benin, Ethiopia, Nigeria, and Senegal (Table 1). Three surgical specialties were explored: neurosurgery [20,23], plastic surgery [18,21,22], and urology [24] (Table 1).

Two teams were further trained in neurosurgery: Vietnamese surgeons performed 15 endoscopic third ventriculostomy with choroid plexus coagulation using Virtual Interactive Presence in Augmented Reality (VIPAR) via iPads in the operating room (OR) [20]. Plus, Tanzanese surgeons performed three open deformity scoliosis corrections using Vuzix smart glasses and Help Lightning software (Table 2). Three teams further trained in plastic surgery: a Palestine surgeon performed a Y-V advancement flap and Z-plasties using Proximie software via a smartphone/camera in the OR. Peruvian surgeons performed 17 cleft and lip reconstructions using Proximie software and Help Lightning software via a tablet in the OR. Mozambique surgeons performed 12 reconstructive surgeries using Google glasses and XpertEye software. The surgeries included facial scar revisions, hand reconstructions with diverse flaps, and lower extremities scar revision with soft tissue coverage with multiple flaps and graft placement (Table 2). One team was further trained in urology: Beninese, Ethiopian, Nigerian, and Senegalese surgeons performed 14 percutaneous nephrolithotomy (PCNL) and urethral reconstructions using Proximie software via mounted camera in the OR or Vuzix smart glasses (Table 2).

**Table 2:** Summary of surgical specialty, procedures, and technology used in the six studies examined VIPAR: Virtual Interactive Presence in Augmented Reality; FTSG: Full-thickness skin graft; STSG: Split-thickness skin graft; LE: Lower extremities; PCNL: Percutaneous nephrolithotomy; W/: with; Post: posterior

Specialty	Study	Mentee	Volume	Procedure	Technology
Neurosurgery	Davis et al. 2016 [17]	Vietnam	n = 15	Endoscopic third ventriculostomy with choroid plexus coagulation	VIPAR: iPad
	Sommer et al. 2022 [21]	Tanzania	n = 3	Scoliosis correction: open deformity correction	Vuzix smart glasses and Help Lightning software
Plastic surgery	Greenfield et al. 2018 [19]	Palestine	n = 1	Hand reconstruction: five flap Y-V advancement and Z-plasty	Proximie software: mentor-tablet; mentee smartphone/camera
	Vyas et al. 2020 [20]	Peru	n = 17	Cleft/lip reconstruction: 14 unilateral and three bilateral	Proximie software and Help Lightning software: tablet
	McCullough et al. 2018 [18]	Mozambique	n = 12	Face: lower eyelid w/eyelid eversion, eyebrow, check scar revision, multiple W-plasties, FTSG. Hand: tendon lengthening and coverage w/post. Tibial artery perforator propeller flap, STSG. Multiple finger reconstruction with multiple random cross-finger flaps and FTSG. LE: scar revisions, STSG. Reverse sural flap for soft tissue defect of heel and STSG. Multiple soft-tissue coverages with antegrade cross-leg sural flap	Google glasses and XpertEye software
Urology	Dominique et al. 2023 [22]	Benin, Ethiopia, Nigeria, Senegal	n =14	PCNL and urethral reconstruction	Proximie software: mounted camera or Vuzix smart glasses

Discussion

Subspecialty Development Using AR Technologies

Concerning urology, Dominique et al. mentee surgeons perform PCNL and urethral reconstructions in Benin, Ethiopia, Nigeria, and Senegal, where there is a high incidence of benign prostatic enlargement, prostate cancer, and urethral stricture disease [24]. Mentees reported AR technology should not replace in-person training; however, it alleviated some foreign mentors' travel and logistical costs, allowing for more frequent training. Additionally, mentee surgical skills were successfully improved with AR training. However, they reported that all cases in the study had connection issues, yet the procedures were successful due to the experience of the trained mentee urologists [24]. Around 75% of mentee surgeons felt that AR training was inferior to in-person training and could not replace its value [24]. However, these relationships are a step forward in closing the worldwide surgical gap, particularly in locations that lack access to safe, timely, and affordable surgical, obstetric, and anesthesia care [25].

The focus of plastic surgery was reconstructive surgery. Vyas et al. performed cleft lip reconstructive surgeries in Peru. They concluded that AR was an effective way to accelerate the transfer of surgical knowledge and improve the continuity of overseas partnerships [22]. Additionally, Greenfield et al. performed a Y-V advancement flap hand reconstruction post-bomb blasting in Palestine. Greenfield et al., in concordance with Vyas et al., stated that AR utilization was not only cost-effective but also reproducible in dispersing the expertise of specialists to reach LMICs [18]. McCullough et al. mentee performed reconstructive surgeries in different facial, hand, and lower extremities [21]. A recent study found that more than 60% of districts in Mozambique needed facilities equipped with ORs, which excludes 44.9% of their population from adequate and timely access to surgical needs of any kind, including obstetric surgeries or complications [26]. McCullough et al. found Mozambique surgeons improved their proficiency in reconstructive surgeries due to the AR training and performed better on-board examinations due to increased knowledge of surgical planning, intraoperative problem-solving, and critical thinking [21]. Safe and affordable surgical care is inaccessible to over five billion people worldwide; any attempt to reduce the gap is significant as the greatest need is by LMICs due to the shortage of surgeons and anesthesiologists [27].

Concerning neurosurgery, Vietnamese and Tanzanian surgeons were trained in endoscopic third ventriculostomy and scoliosis corrective surgery, respectively [20,21]. Davis et al. concluded that VIPAR successfully assisted and guided Vietnamese surgeons in developing new skills in their domains [20]. Sommer et al. found that using AR technology for corrective scoliosis surgery is challenging. They discovered that smart glasses automatically reduced image quality in addition to the internet connection and required the mentee surgeon to hold their head extremely still to avoid misunderstandings about surgical markings [23]. Further, differing time zones also created challenges with 10+ hour time differences between surgeons [21,23]. Overall, mentors suggested and highlighted how imperative it was that the surgeons already possess a functional skill set in the case of technical difficulties, such as loss of internet activity [20,21].

Bidirectional HIC-LMIC-LMIC Relationships

The US and UK are the leading countries in creating pipeline programs to develop AR technologies in surgical fields in LMICs [18,20-24]. Our results show that Vietnam, Mozambique, Palestine, Peru, Tanzania, Benin, Ethiopia, Nigeria, and Senegal have had experiences with US or UK surgical mentors to train in AR technologies (Table 2). Further partnerships between institutions in HICs and LMICs have grown [28], such as in 2011, when Spain, Turkey, and the US helped Tanzania and India treat brain and spinal disorders [29]. Additionally, between 2006 and 2019, Norway assisted Ethiopia in treating spinal disorders using diverse technologies [30]. While these partnerships used different virtual or telemedicine technologies, they successfully expanded surgical education worldwide and could be a gateway to implementing AR technologies to continue assisting LMICs and underserved populations.

Most collaborations to assist this shortage in surgical training have been through HIC-LMIC alliances. However, per our results, an LMIC-LMIC partnership was created when a Lebanese surgeon assisted a Palestine surgeon in hand reconstruction [18]. This research team found no other examples of LMIC-LMIC relationships regarding operative training or assistance. Overall, the mentoring from HICs and LMICs should continue as the gap in surgical access is still very prevalent in LMICs, and there is a shortage of surgical specialists worldwide, greatly impacting LMICs [31].

Implementation of AR Technologies

Another consideration is the cost and execution of AR technologies for hospitals in LMICs. Davis et al. projected the total costs of establishing VIPAR for one year at $14,930.39 [20]. The market price of the Proximie system and Vuzix smart glasses is $17,000 and $1300-2500 per year. However, both technologies were donated to Medi Tech Trust UK Charity in the Dominique et al. study [24] and found cost-effective in the Greenfield et al. case study [18]. For countries that cannot afford these technologies' costs, it may be beneficial to establish foundations to raise funds and collect donations to implement them for LMIC hospitals. McCullough et al. acknowledge that the $6,990 price of the yearly contract for wearable hardware and Expert Eye operating platform is not insignificant in LMICs, while it is much less than the cost of importing a team of surgeons [21]. Vyas et al. reported hardware costs of $2200 and AR software license costs of $7000 [22]. While these data may be significant per geographical region, the costs of several of these factors vary depending on location and the scarce products or services in the areas.

Future studies may find it beneficial to utilize long-term patient outcomes, surgeon skill retention, larger cohorts of trainer-trainee pairs, and compare varying types of AR, including standing AR consoles and eyewear [21,24]. The cost-effectiveness of telemonitoring in LMICs is an entirely separate topic that must be explored in future studies while comparing varying methods of AR to bringing in a team of surgeons from a HIC [21]. Most surgeons also wear surgical loupes, which sometimes interfere with the Google glass headset, so AR technology specifically for surgeons must be further modified [21]. Future research could focus on modifications to the smart glasses to use optical filters over the camera lenses and other strategies to strengthen internet connection to achieve more precise image quality under surgical lighting and reduce time lag [23].

## Conclusions

AR defined as audio and visual live feedback has been used in LMICs in the surgical field. The main surgical specialties that have developed training using AR are neurosurgery, plastic surgery, and urology. There are various combinations of software using AR in the surgical field. Although costs and connection issues have led to some challenges in the studies examined in this review, the integration of AR in surgical training represents a promising start toward tackling the enduring challenges posed by limited surgical capacity in LMICs. This innovation has the potential to narrow the global disparity in surgical proficiency, marking a significant step forward in addressing these critical issues.
